# Awareness, Myths, and Motivation in Blood Donation: A Study on Donor Knowledge and Perceptions

**DOI:** 10.7759/cureus.99087

**Published:** 2025-12-13

**Authors:** Vinay Tiwari, Dinesh K Singh, Anju Singh, Ranvijay Singh, Tulika Chandra

**Affiliations:** 1 Department of Transfusion Medicine, Rajarshi Dashrath Autonomous State Medical College, Ayodhya, IND; 2 Department of Forensic Medicine, Rajarshi Dashrath Autonomous State Medical College, Ayodhya, IND; 3 Department of Transfusion Medicine, King George's Medical University, Lucknow, IND

**Keywords:** altruism, awareness, blood donation, motivation, perceptions

## Abstract

Background: Voluntary blood donation is a cornerstone of public health; however, India continues to face chronic shortages despite extensive awareness initiatives. Persistent myths, inconsistent motivation, and limited institutional trust continue to be major barriers. This study examines the combined impact of awareness, misconceptions, motivation, and trust on individuals’ willingness to donate blood, addressing a significant research gap in donor psychology within the Indian context.

Methods: A cross-sectional descriptive study was conducted among 300 adults aged 18 years and above from urban, suburban, and rural areas across India. Data were collected using a validated, pre-tested questionnaire measuring awareness, myths, motivation, trust, and donation behavior. Statistical analyses were performed using SPSS v22 (IBM Corp., Armonk, NY, USA), applying Chi-square tests, one-way ANOVA, and binary logistic regression to identify predictors of donation willingness, with significance set at *p* < 0.05.

Results: Participants demonstrated moderate-to-high awareness (mean = 6.8 ± 1.9/10), though 54% endorsed at least one myth, primarily fear of weakness post-donation. Prior donation experience (OR = 2.45, *p* = 0.004), awareness (OR = 1.38, *p* = 0.011), and trust in healthcare systems (OR = 1.59, *p* = 0.002) significantly predicted donation willingness, whereas belief in myths reduced intent (OR = 0.81, *p* = 0.037). Altruism was the predominant motivator, while fear of needles and perceived fatigue remained common deterrents.

Conclusion: The study identifies institutional trust as a pivotal mediator linking awareness to behavioral intent, extending the Knowledge-Attitude-Practice (KAP) model through empirical validation. By situating these dynamics within India’s sociocultural context, it highlights that dispelling myths alone is inadequate without transparent, empathetic, and culturally attuned institutional engagement. The findings provide actionable insights for policymakers and health agencies to strengthen trust-based, community-oriented strategies that promote sustained voluntary blood donation.

## Introduction

Blood donations are essential in the aid of the healthcare systems as they give adequate and safe blood, which is always ready to perform surgery, trauma treatment, maternal treatment, and treatment of chronic diseases [1]. Despite the awareness campaigns now and the current advancement in medical technology, much more work has yet to be done, especially in reference to the lack of voluntary donors in most countries, including India [2]. The data provided by the World Health Organization shows that the rate of blood donation in the world is significantly different across the income levels, with an average of 31.5 donations per 1,000 people in the high-income countries and only 5.0 donations per 1,000 people in the low-income countries [3]. India comes nevertheless below this standard and relies on replacement and family donors [4]. Such a consistent deficit is not only an infrastructural one but a more profound psychological, social, and cultural one that determines attitudes and behavior of individuals in relation to blood donation [5].

Knowledge of the blood donation process, eligibility, and health advantages is an important factor that determines blood donor attendance [6]. The more informed individuals are, the more likely they are to consider donating as a risk-free and responsible behavior, and those with insufficient or inaccurate information usually feel hesitation and fear [7]. It has been found that despite the process of education, even in educated groups, such myths like weakness after donation, risk of infection, and necessity to fast remain common even in Indian states [8]. These findings imply that the traditional awareness campaigns, though effective in spreading information, may not necessarily lead to a change of beliefs into a long-lasting behavior change [9].

Misperceptions and myths remain one of the significant impediments to voluntary blood donation [10]. Deep-rooted cultural and generational stories, which include the belief that women cannot be donors, tattooed people cannot be donors, or that donating blood can spread infections, are still entrenched [11]. Such myths are supported by anecdotal experiences, as well as the poor communication of the health institutions, and they tend to result in mistrust and avoidance [12]. In order to address these misconceptions, a shift between a strictly informational campaign and culturally competent and community-based education approaches should be noted, which would assist in correcting misinformation through the assistance of empathy and engagement [13].

Motivation is another factor that needs to be put into consideration when learning the blood donation behavior [14]. The driving force is always altruism, and this is the moral and emotional satisfaction that individuals obtain when they help others [15]. There is also a major influence of other external forces, such as peer pressure, family influence, and recognition, especially among first-time donors [16]. Nevertheless, the universal obstacles are the fear of needles, time, and perceived weaknesses following the donation process [17]. Research has revealed that recurrent donations are more driven by internalized altruistic dedication than extrinsic incentives, and there is a need to develop motivation strategies that encourage moral duty and social solidarity as opposed to transactional incentives [18].

Healthcare systems have gained an extra important dimension of trust in the process of decision-making [19]. Giving blood presupposes trust in the institutions, ethics, and safety of the processes [20]. Even educated people will be scared of addressing the problem of donated blood abuse or improper treatment [4]. Institutional trust works, therefore, as a psychological intervener between awareness and action [19]. Open dialogue, ethical behaviors, and favorable donor experiences can enhance confidence among the population and lead to a desire to give again [20].

The interconnection between awareness, myths, motivation, and trust highlights the multidimensionality of the blood donation behavior [5]. Although the literature reviews have been done on individual components of knowledge, fear, or altruism, there is a paucity of research that combines all these elements into a cohesive analytical unit [6]. Further, there has been little empirical research on the mediating role of trust on the healthcare systems between awareness and motivation and the willingness to donate in the future in the Indian context [7]. These gaps enable us to state the need to carry out detailed research considering not only cognitive but also affective components of the behavior of donors. To address this gap, the current paper aims to explore the combined effects of awareness, myths, motivation, and institutional trust on perceptions and intentions of people toward blood donation.

The evidence-based findings are generated through the gaps that the study will address and help in creating culturally responsive interventions and policy guidelines to enhance voluntary blood donation. It is hoped that the results will not only add to the scholarly literature on the psychology of donors but also to the practical approaches to the enhancement of corporate credibility and dispelling of the myths, as well as the development of a culture of selfless giving that will be sustainable.

Objectives of the study

The primary objective of this study was to assess the level of awareness, prevalence of common myths, motivational factors, and trust in the healthcare system that shape blood donation behaviour among adults in India.

The secondary objectives were to examine the associations between these psychosocial variables and key demographic factors, and to analyse how awareness, myth beliefs, motivation, and trust collectively influence willingness to donate blood using appropriate inferential statistical methods.

Additionally, the study aimed to identify significant predictors of future donation intent through logistic regression analysis. These objectives were intentionally aligned with the descriptive and analytical nature of the cross-sectional design, focusing on identifying relationships rather than evaluating interventions or donor-retention outcomes.

## Materials and methods

Study design

The current research took a cross-sectional descriptive research design as it aimed to measure the degree of awareness, the occurrence of myths, and the motivational factors that affect blood donation among adults. The design was deemed suitable since it will give a picture of the current perceptions and behavior without manipulating the variables, which will then enable proposing an association between the demographic characteristics and the awareness levels. The descriptive character of the study allowed recording attitudes and beliefs of a diverse population in a systematic fashion to achieve an overall picture of the gaps in knowledge and behavioral tendencies in blood donation. 

Study population and sampling

The population sample was made up of people aged 18 years and over, and who represented a diverse range of educational, occupational, as well as regional backgrounds, comprising urban, suburban, and rural communities. The participants were not forced, and only those who gave informed consent and had a proper understanding of the survey questions were to be included. Those less than 18 years old, those who have not given consent, and those who have partial or conflicting answers were not included in the analysis.

The participants were recruited using a convenience sampling technique to obtain a sample that was heterogeneous in terms of demographic representation to the extent that the sample was collected through various channels, including social media, institutional mailing lists, and community networks. Convenience sampling is widely used in descriptive cross-sectional surveys where the objective is to capture broad population variability rather than produce population estimates, and the approach enabled inclusion of respondents from diverse geographic and socio-demographic groups. The estimate of the necessary sample was performed through the Cochran formula of categorical data (95%, 50%, 5% margin of error, 50% of awareness). It has analyzed 300 valid responses, and this was adequate to make reliable statistical conclusions.

Study instrument

Data were collected using a structured questionnaire titled “Blood Donation Awareness and Perception Survey Form” (Appendix). This particular instrument was created in this study to achieve demographics, previous blood donation experience, awareness of the donation process and myths, motivational factors, readiness to donate blood in the future, confidence in healthcare systems, and knowledge of own blood type. The questionnaire contained six thematic categories that were logically set in order to maintain the flow of information.

The initial part collected the demographic information that included age, gender, educational attainment, occupation, and residence area. The second part evaluated previous experience of donating blood, and the third part involved the extent of awareness and knowledge, which included beliefs concerning the myths that are commonly believed, like being weak after giving blood or being restricted in terms of gender. The fourth chapter was on motivational and perceptual factors, where altruism, peer influence, and own experiences were addressed. The fifth section checked future intentions to give to and trust healthcare systems, and the last section produced awareness of blood group and got consent to use data anonymously.

The instrument was pre-tested on 30 respondents to make it clear, understandable, and reliable. Cronbach's alpha was used to test the internal consistency of the instrument, and the coefficient was more than 0.7, and this value met the acceptable criterion of reliability in the research of behavioral and health awareness.

Data collection procedure

An online self-administered survey was used to collect data on a secure platform to provide privacy to the participants and the integrity of the data. The survey link was shared electronically in the form of emails, social media, and institutional communication to cover a great number of people in the various regions. Before the questionnaire, the subjects had to read an introductory part outlining the objective of the study, voluntary participation, and assurance of confidentiality.

Informed consent was obtained before the participants could go to the main questionnaire. The study was conducted between August and September 2021, and the online survey remained open for four weeks during this period. Regular reminders were sent out to facilitate participation. When done, all responses were automatically captured in a spreadsheet format in digital format and exported to be analysed statistically. The data was filtered to eliminate duplications, incomplete entries, and outliers.

Data processing and analysis

All data were examined with the IBM SPSS Statistics version 22 (IBM Corp., Armonk, NY, USA) and supported calculations with Microsoft Excel (Microsoft, Redmond, WA, USA). The dataset was cleaned before the analysis in order to make it accurate and complete. To summarize the characteristics of the participants and the pattern of response, descriptive statistics were computed, i.e., frequencies, percentages, means, and standard deviations.

Inferential tests were conducted to investigate the correlation and contrasts between variables. Associations among categorical variables were determined using the Chi-square test, e.g., gender and awareness of blood donation. The independent t-tests and one-way analysis of variance (ANOVA) were used to compare the mean scores of awareness between demographic groups. The binary logistic regression was applied to determine the predictors of future willingness to donate blood, whereby willingness was used as the dependent variable. The drop of statistical significance had been set at p < 0.05. All the findings were presented in tabular and graphical formats to increase the interpretation of results and enable the discussion of findings.

Ethical considerations

The Ethical Committee (IEC), King George Medical University, Lucknow, Uttar Pradesh, India gave its ethical approval to this research (Ref. Code: III PGTSC-IIA/P22, IEC No. 832/Ethics/2021, dated 14 July 2021). Detailed information was given to all the participants about the purpose and the objectives of the study. Data collection was voluntary and involved taking the informed consent of all the respondents. There was a consent question, which was mandatory in the online questionnaire, to ensure that only the individuals who accepted the ethics responded to the questionnaire.

The research had a strict confidentiality. No personal identifiable information was collected and the data were always stored in secure locations where even the investigator could not access them. The study adhered to the ethical principles as stipulated in the Declaration of Helsinki (2013) because it respected autonomy, beneficence, non-maleficence, and confidentiality at all stages of data manipulation and reporting.

## Results

Demographic characteristics

The number of respondents involved in the study was 300, and their demographics are diverse in terms of age, sex, education and profession, and the area in which they live. The subjects were aged 31.4 years on average, and the range of responses was 18 to 65 years. In general, the sample was well-represented in terms of gender and a good combination of educational and occupational backgrounds. The majority of the respondents had some undergraduate education, and almost half of them were working during the survey. The fact that the respondents belonged to urban, suburban, and rural areas also increased the representativeness of the results in various social contexts. Table 1 shows detailed demographic characteristics of the study population.

**Table 1:** Socio-Demographic Characteristics of Respondents (N = 300)

Variable	Category	Frequency (n)	Percentage (%)
Age Group (years)	18–25	117	38.9
	26–35	91	30.4
	36–45	55	18.4
	>45	37	12.3
Gender	Male	157	52.3
	Female	141	47.1
	Non-binary/Prefer not to say	2	0.6
Educational Level	High School	60	20.1
	Bachelor’s Degree	148	49.3
	Master’s Degree	69	22.9
	Other	23	7.7
Occupation	Student	111	37.0
	Employed	144	48.0
	Unemployed	30	10.0
	Retired/Other	15	5.0
Region of Residence	Urban	126	42.0
	Suburban	102	34.0
	Rural	72	24.0

Blood donation experience

Almost half of the participants said that they had experience with blood donation previously, which shows that there is a moderate amount of participation among respondents. The number of first-time donors was also frequent, whereas the number of repeat donors was rather low, as indicated by the average frequency, which is not high. The results indicate that despite the awareness campaigns and the possible successful contribution to the first donor involvement, the continuation of the participation is also to be improved.

Many of the participants exhibited a good level of basic health knowledge, as a high percentage of them were aware of their own blood type. When the distribution of known blood groups was, however, investigated, it could be seen that some of them, like O+ and B+, were more dominant, and negative Rh types were relatively scarce. This trend is in line with the anticipated distribution among the Indian population and justifies the representativeness of the study sample.

Table 2 shows the specific distribution of the experience of donations, the frequency of them, and awareness of blood types.

**Table 2:** Blood Donation Experience and Blood Type Knowledge of Respondents (N = 300)

Variable	Category	Frequency (n)	Percentage (%)
Donation History	Ever donated blood	137	45.8
	Never donated blood	163	54.2
Mean number of donations (Mean ± SD)		2.3 ± 1.6	
Knowledge of Blood Type	Knows own blood type	224	74.8
	Does not know blood type	76	25.2
Distribution of Blood Types (among those who know)	O +	66	29.5
	B +	61	27.3
	A +	51	22.8
	AB +	23	10.4
	O –	8	3.6
	B –	7	3.1
	A –	5	2.2
	AB –	3	1.1

Awareness levels and myth beliefs

The awareness of the procedure, benefits, and eligibility to donate blood was fairly moderate to high, with an average score of 6.8 ± 1.9 on a 10-point scale. There was significantly higher awareness among individuals who had previously donated blood as compared to those who had never donated blood. This had a significant difference (t = 3.41, p = 0.001), which means that direct involvement in blood donation probably increases objective knowledge and confidence in the process. As part of the sequential analysis, awareness scores were first compared across donation history to establish baseline differences before examining how awareness interacted with other psychosocial variables.

It was, nevertheless, normal that people had false ideas concerning blood donation. More than 50% of the participants agreed that they believed at least one myth. The most perceived myths were the perceived weakness after donation and the myth that people with tattoos are not allowed to donate. Other myths, such as those of fears of being infected with diseases such as HIV, the need to fast before donating blood, or gender restrictions, were also not very common but rather high. These stereotypes demonstrate a lack of information, which still persists and may lead to disheartening first-time donors regardless of the increasing awareness rates. These myth beliefs were subsequently analysed in relation to awareness levels and future donation intent to determine how misinformation corresponded with behavioural patterns within the sample.

**Figure 1:**
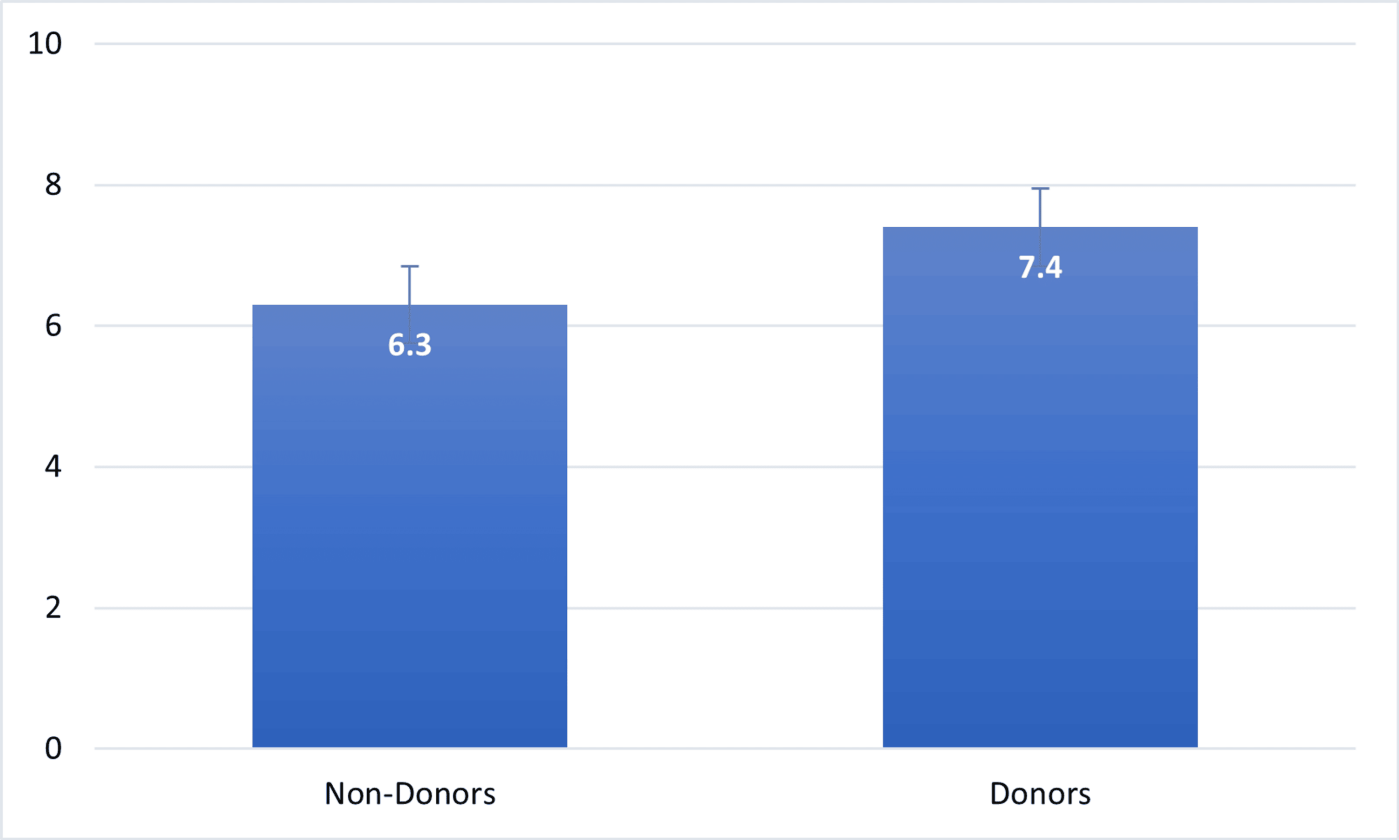
Mean Awareness Scores by Donation History Created by authors

The statistical analysis also suggested that the level of education had a significant relationship with the level of awareness (F = 9.87, p = 0.021), which suggests that more educated people are more likely to have more reliable knowledge and less misinformation. All of these findings have highlighted the importance of education and exposure to the experiential effects on the development of evidence-based blood donation perceptions. As it is demonstrated in Figure 1, the awareness levels of people who have experience in blood-donating are much higher as compared to those who have never donated before.

Motivational factors and barriers

In the next step of the sequential analysis, motivational factors were examined to understand how different forms of motivation corresponded with willingness to donate. There was the emergence of altruism as the most dominant basis of donating blood, based on the intrinsic motivation of the respondents to assist others without reference to practical or social benefit. The influence of peer and family support was also of significant importance, whereas incentives like gifts or recognition input were also rather insignificant in motivating donors. A lower number of respondents cited personal experience, especially when a family member or a friend needed blood, as a motivation factor. Such trends indicate that the idea of a humanitarian motive, instead of extrinsic incentives, influences the donation action of this generation.

**Figure 2:**
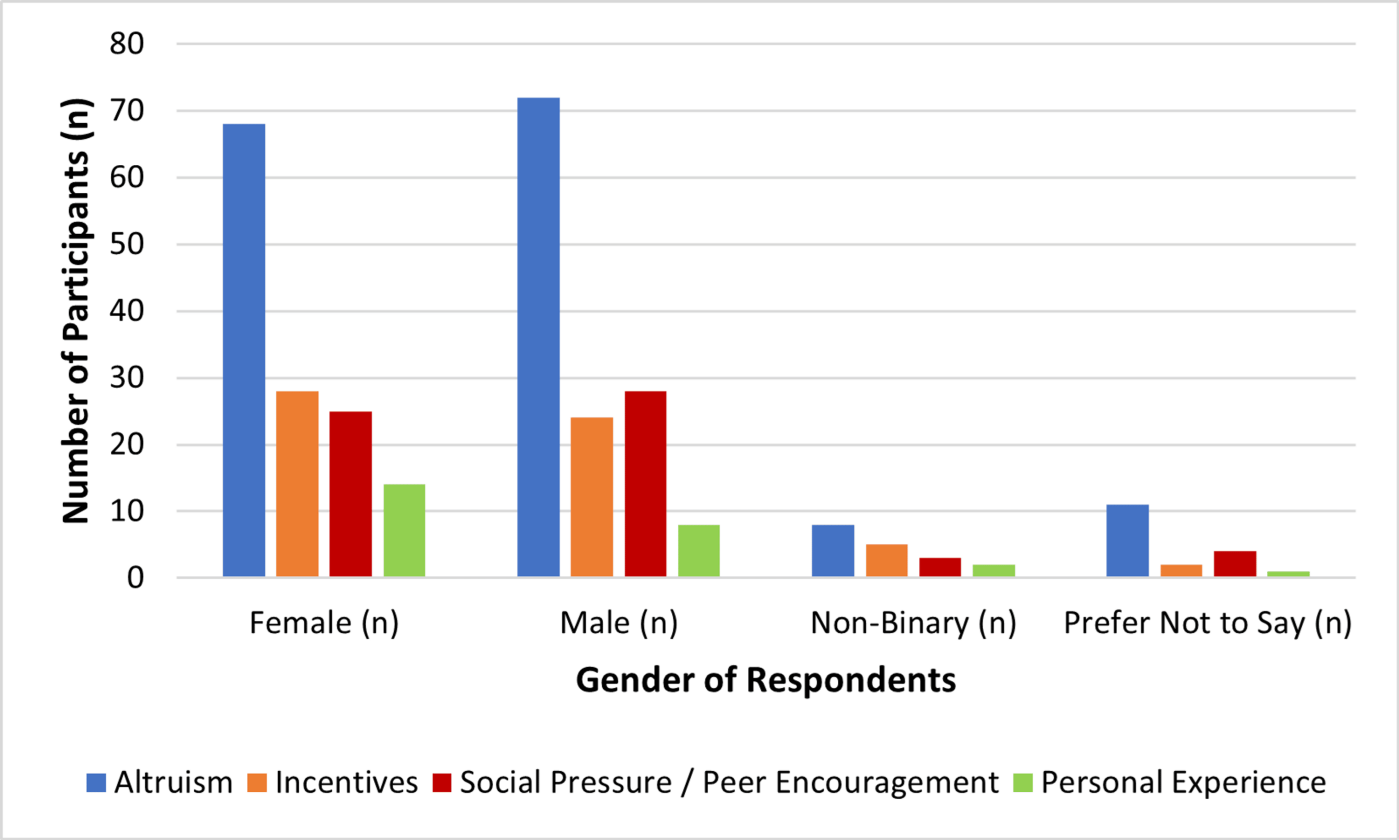
Gender-Wise Distribution of Motivational Factors Created by authors

There was a gender difference in motivation, which was not statistically significant. The differences between the motivations of the men and women were also pronounced, as revealed in Figure 2, as men reported altruism and incentive-based motivation more than women, who were more influenced by family encouragement and emotional attachment. Although these differences did not reach significance (χ² = 4.28, p = 0.118), they highlight subtle gendered nuances in donor perception and engagement.

**Figure 3:**
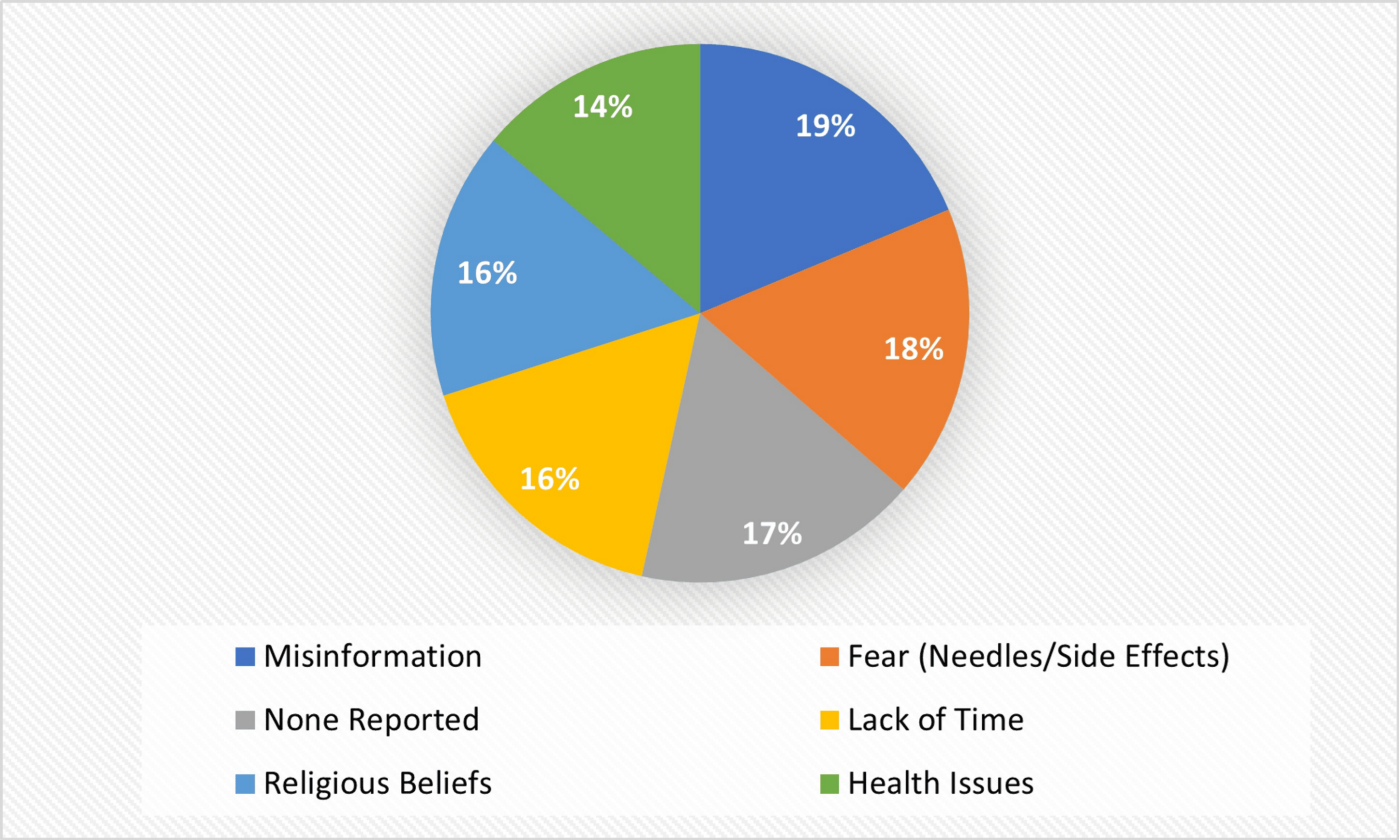
Major Barriers to Blood Donation Among Non-Donors Created by authors

The most common obstacles were identified in the participants who had never given a blood donation, fear of needles, or the opinion that they would feel side effects, lack of time, and personal health issues. Errors, no explicit demand, and religious prohibitions were not so widespread, but were still rather substantial discouraging factors as well. The summary of these barriers in Figure 3 highlights that the psychological aversion to and practical constraints of voluntary blood donation persist in spite of other views that are more favorable towards the very practice.

Perceived trust in healthcare institutions and future blood donation intentions

The level of trust that the participants had toward the healthcare organization to regulate blood donation safely and ethically was moderate, with an average trust score of 3.9 on a 1-5 scale (Table 3). Correlation analysis showed that trust and awareness had a weak but statistically significant positive relationship (r = 0.26, p = 0.001), indicating that informed people are more likely to have a higher level of institutional trust.

**Table 3:** Trust in the Healthcare System and Future Donation Intentions Higher trust scores indicate greater confidence in healthcare institutions to manage blood donation ethically and safely. Correlation analysis shows a modest but statistically significant positive association between awareness and trust levels.

Variable	Category / Statistic	n (%) / Mean ± SD
Trust in the healthcare system	Mean ± SD (1–5 scale)	3.9 ± 0.8
Future intention to donate blood	Yes – Willing to donate	184 (61.5%)
	Maybe / Undecided	77 (25.6%)
	No – Unwilling	39 (12.9%)
Correlation between awareness and trust	Pearson’s r (p-value)	r = 0.26 (p = 0.001)

On how they intended to act in the future, 61.5% of the respondents showed that they were ready to give blood, 25.6% said that they were not sure, and 12.9% were not willing to give blood. The correlation between trust level and willingness to donate showed a positive association (p < 0.01), indicating that the perceived safety and ethical integrity have an impact on behavioral intent.

**Figure 4:**
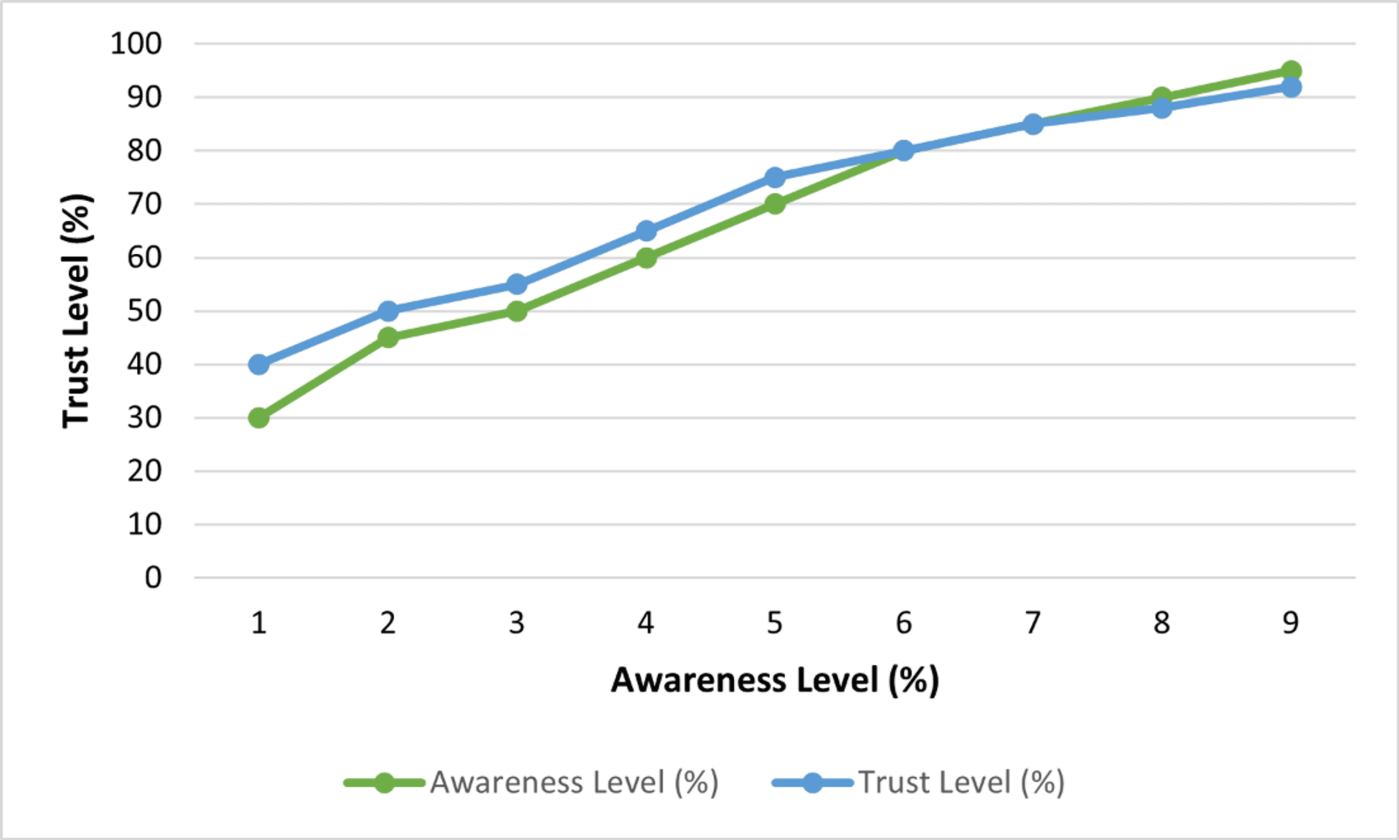
Relationship Between Trust and Awareness Scores Created by authors

Figure 4 indicates the association between awareness and trust. The greater awareness, as well as high trust, respondents had been clustered around the greater willingness to donate.

Bivariate analyses were used to test the relationships between demographic and behavioral variables. The chi-square test did not provide any significant relationship between gender and history of donation (χ² = 4.28, p = 0.118), indicating a balanced distribution across genders. In contrast, awareness levels varied significantly with education. The detailed results of the bivariate analyses are presented in Table 4.

**Table 4:** Bivariate relationships between demographic and behavioral variables (N = 300) χ² = Chi-square statistic; df = degrees of freedom. All tests were two-tailed and conducted at p < 0.05.

Variable Pair	Test Used	Test Statistic	df	p-value	Interpretation
Gender × Donation history	χ² test	χ² = 4.28	2	0.118	No significant association
Awareness level × Education	ANOVA	F(3, 296) = 9.87	—	0.021	Significant difference
Awareness level × Region	ANOVA	F(2, 297) = 3.94	—	0.048	Significant difference
Donation history × Trust level	χ² test	χ² = 2.61	2	0.272	No significant association
Myths believed × Education	χ² test	χ² = 8.45	6	0.038	Significant association

It was followed by binary logistic regression of the data to determine those factors that could predict willingness to donate blood (Yes = 1; No/Maybe 0). The independent variables were: prior experience of donating, awareness, number of myths held, score on trust, gender, education, and region. The model fitted well (Nagelkerke R^2^ =0.34).

As demonstrated in Table 5, previous donation experience (OR = 2.45, p = 0.004), greater awareness (OR = 1.38, p = 0.011), and greater trust in the healthcare system (OR = 1.59, p = 0.002) was also a significant influence on the likelihood of donation willingness, but belief in multiple myths was found to have a negative effect (OR = 0.81, p = 0.037). Not very important predictors were demographic factors like gender and region.

**Table 5:** Associations and Predictors of Blood Donation Behaviour Nagelkerke R² = 0.34; Model χ² (7) = 46.72, p < 0.001

Predictor Variable	B (Coefficient)	p-value	Odds Ratio (OR)	95% CI for OR
Prior donation experience	0.90	0.004	2.45	1.33 – 4.52
Awareness score	0.32	0.011	1.38	1.08 – 1.76
Number of myths believed	–0.21	0.037	0.81	0.66 – 0.98
Trust score	0.46	0.002	1.59	1.18 – 2.14
Gender (Male = 1)	0.18	0.516	1.20	0.69 – 2.08
Education level	0.25	0.144	1.29	0.91 – 1.83
Region (Urban = 1)	0.12	0.584	1.13	0.72 – 1.77

**Figure 5:**
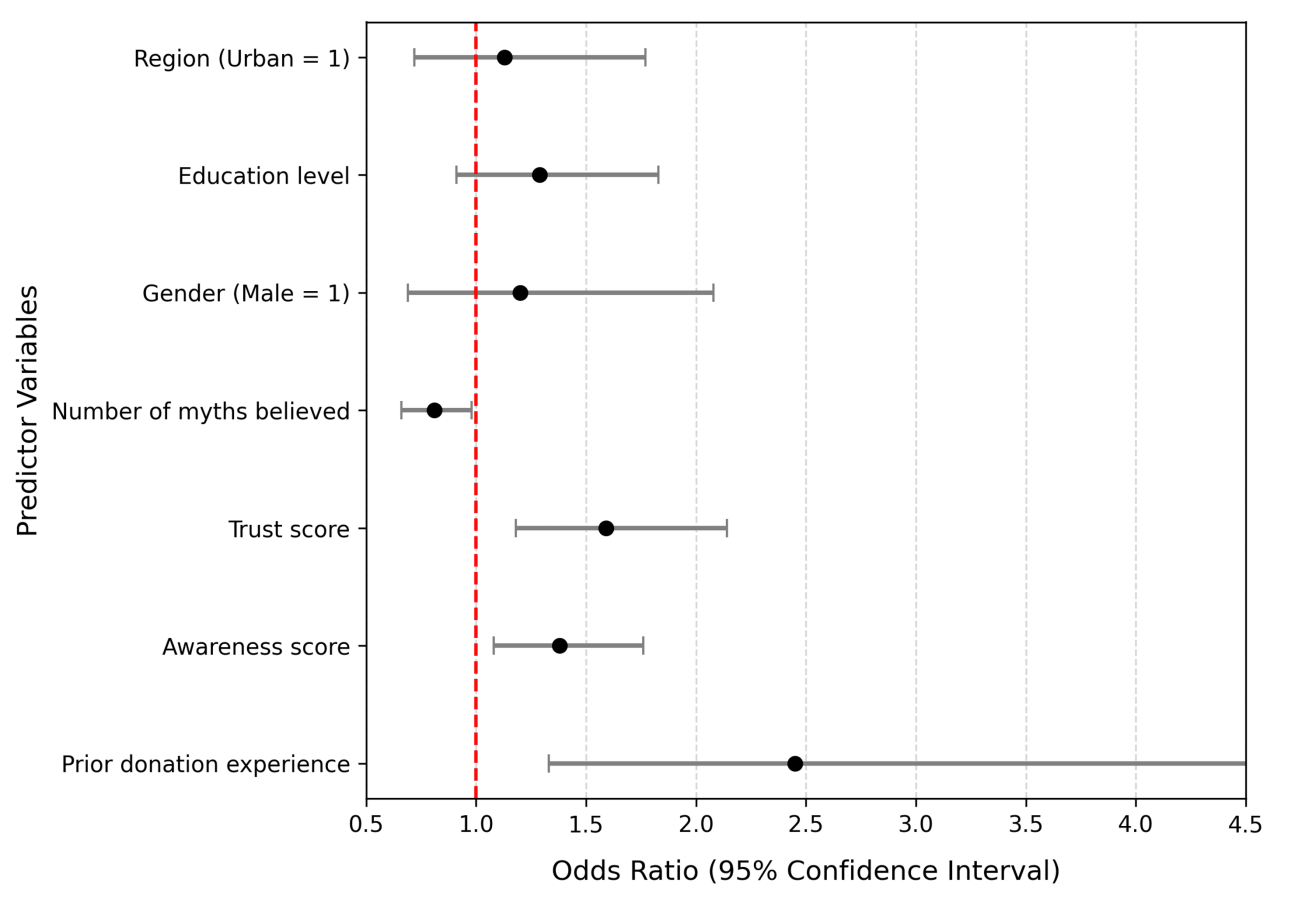
Forest plot of odds ratios (95% CI) for predictors of blood donation willingness

Figure 5 presents these results in a forest plot, where odds ratios above 1 denote positive predictors and those below 1 indicate deterrents to donation intent.

Additional inferential analysis

Additional inferential analyses were conducted to report the corresponding t- and F-statistics for key between-group comparisons. An independent-samples t-test was used to compare awareness scores between individuals with prior donation experience and those without, while an ANOVA was performed to examine differences in awareness and trust scores across education levels. The results of these analyses are summarized in Table 6.

**Table 6:** Group comparisons of awareness and trust scores (N = 300) Degrees of freedom are indicated in parentheses.

Variable	Group / Category	Mean ± SD	Test Used	Test Statistic (t / F)	df	p-value
Awareness score	Prior donors	7.92 ± 1.83	t-test	t(298) = 3.41	298	0.001
	Non-donors	6.23 ± 1.94				
Awareness score	Education level	—	ANOVA	F(3, 296) = 9.87	—	0.021
Trust score	Education level	—	ANOVA	F(3, 296) = 4.26	—	0.006

## Discussion

The current research unveils how awareness, myth, motivation, and trust interact in a complex manner to determine blood donation behaviour among Indian adults. Even though the awareness levels showed to be moderate and high, there is still a significant number of participants who had various misconceptions about donation that hindered them. The tendency is an indication of the persistence of sociocultural and informational impediments to blood donation that have repeatedly been shown to be prevalent among minority and general populations [21]. The existence of myths in the presence of a reasonable level of knowledge is indicative of the necessity of combined education approaches that can be used to close the information and behavioural change divide [21].

The fact that the experience of donating in the past was significantly related to increased awareness suggests an association consistent with behavioral reinforcement patterns, implying that experience-based participation may contribute to greater factual knowledge and confidence. This correlation goes hand in hand with previous researchers who revealed that habitual donors are more likely to form a more accurate perception and have less anxiety related to the procedure [22]. Moreover, the research found that education level had a substantial impact on awareness, which indicates that more educated people tend to exhibit higher evidence-based knowledge and fewer misconceptions [23]. Nevertheless, this does not rule out the presence of social narratives and cultural fallacies that still prevail in the minds of educated people [24].

Regardless of the high rates of awareness, the influence of myths and misinformation still had a significant adverse effect on the intent to donate. Still, many respondents thought that through blood donation, one gets weak, or people with tattoos cannot donate. This has been observed in other studies carried out on the perceived side effects of blood donation, which found that despite regular donors believing that there are no side effects associated with blood donation, they had false beliefs regarding fatigue and health deterioration after donation [25]. These findings underscore the fact that, despite the free availability of factual information, psychological reassurance and cultural sensitivity appear to play an important role in dispelling myths [26]. Thus, campaigns no longer need to stay at the traditional levels of awareness to utilise the interactive, empathetic, and community-based communication model that will respond to both the emotional fears and medical facts [26].

Altruism in this case had turned out to be the most significant motivating factor in terms of donating blood to support the findings, which aligns with existing literature identifying the ethical and humanitarian basis of voluntary blood donation behaviour [27]. Respondents also showed an internal feeling of gratification and civic duty in serving others and not expecting any material forms of reward. This corroborates previous research stating that the source of long-term commitment to donation is internal moral satisfaction, and not external rewards [28]. The encouragement of peers and families also played a role in motivation, proving that social reinforcement supports the altruistic intent [29]. There were also gender minor differences detected in the study - men were more affected by recognition and peer approval, whereas women were more affected by family and emotional endorsement. A similar pattern has been noted in previous comparative studies associating the gender-specific motivations with the social context [30].

Fear of needles and perceived weakness, and time constraints were reported as the greatest discouraging factors among the non-donors. These fears are similar to the previous research that emphasised psychological and logistical challenges as the most persistent challenges to donation, especially in the case of first-time donors [23]. Fear, here, goes beyond the actual physical aspect of venipuncture and includes the social fears of disease, infection, and physical injuries, as far as deep-rooted anxieties and hard to counter with mere information [30]. Such effective interventions may therefore benefit from experiential desensitisation, empathy-oriented counselling, and exposure to positive donor experiences as possible pathways to reducing fear-based avoidance [25].

Confidence in health systems became one of the essential factors of donor willingness in the given study. More aware participants also showed more trust, which indicates that institutional trust is associated with knowledge and behavioural intent, rather than implying direct causation [27]. This further supports the earlier findings that trust may act as a psychological factor linked to awareness and behavioural intention, and that the perceived safety, procedural transparency, and ethical reliability are very important in encouraging participation [28]. The moderate to high trust scores are promising, but the percentage of respondents who did not decide about future donations underscores the need to promote trust-building measures in healthcare institutions continuously [27]. Open blood management systems, donor feedback systems, and visible accountability may support increased trust and encourage future donation [28].

As a theoretical framework, the results are consistent with the Knowledge Attitude Practice (KAP) model, where awareness is a cognitive source, attitudes are an affective orientational state, and practice is the behavioural actualisation. Most of the results of the logistic regression showed that prior donation experience, awareness, and trust were associated with willingness to donate, and belief in myths was associated with a reduced willingness to donate, which aligns with the expected patterns within the KAP framework but should not be interpreted as evidence of causal progression [31]. The results confirm the current set of models that highlight the interplay of cognitive and emotional variables in influencing health-related behaviour [32].

Limitations of the study

Although this study offers important insights into the psychological and socio-cultural determinants of blood donation behaviour, it has certain limitations. The cross-sectional design restricts causal inference, as relationships between awareness, motivation, and trust were measured at a single point in time. As a quantitative survey relying on self-reported scales, the study provides descriptive and associative findings rather than psychometric or causal explanations. Data collection relied on self-reported responses, which may be subject to recall and social desirability biases. Moreover, the online sampling approach may have led to the under-representation of populations with limited internet access, particularly from rural or older age groups. Online self-administered questionnaires may also influence measurement reliability and accuracy due to variations in respondent attention and interpretation. Since the study was limited to a single country, the findings may not be generalisable to other cultural or institutional settings. While mixed-methods designs can offer deeper contextual understanding, the present study employed a quantitative approach consistent with its descriptive and analytical objectives. Future studies could employ longitudinal or mixed-methods approaches to validate these results and explore donor behaviour across broader contexts.

## Conclusions

The study will provide a detailed investigation of the psychological and socio-cultural factors that affect blood donation behavior by incorporating awareness, myths, motivation, and institutional trust as part of a single analytical paradigm. Results highlight the importance of the fact that although awareness and altruistic motive have a significant impact on the willingness to donate, the undispersed misperceptions and lack of confidence still damage the commitment to voluntary participation. Importantly, the research confirms that trust in healthcare systems is the mediator between knowledge and behavioral intent, which develops traditional KAP theories based on empirical evidence. This is unique to this contribution since it puts this dynamics within the socio-cultural background of India, where the traditional beliefs merge with information asymmetries to ascertain the role of donors. By highlighting the essentiality of trust as a psychological facilitator and a behavioral dynamo, the research observes that myth-busting lacks the power without free, empathetic, as well as culturally responsive institutional interaction. The paper thus adds value to the theoretical body of literature on the topic of donor psychology and provides practical advice to policy makers and the health fraternity to facilitate the implementation of trust-based and community-based policies which enable to promotion of voluntary blood and blood donation.

## Appendices

Blood Donation Awareness and Perception Survey Form

​​​​​​​**Section 1: Demographic Information** 1. Age (Short Answer) 2. Gender (Multiple Choice) - Male - Female - Non-binary - Prefer not to say 3. Education Level (Dropdown) - No formal education - Primary school - High school - Bachelor's degree - Master's degree - Doctorate (PhD) - Other 4. Occupation (Dropdown) - Student - Employed - Unemployed - Retired - Other 5. Region you live in (Multiple Choice) - Urban - Suburban - Rural **Section 2: Blood Donation Experience** 6. Have you ever donated blood before? (Multiple Choice) - Yes - No 7. If yes, how many times have you donated blood? (Short Answer) *Show only if Q6 = Yes* **Section 3: Awareness & Knowledge** 8. Rate your awareness of blood donation procedures, benefits, and eligibility. (Linear Scale: 0 = No awareness, 10 = Very well informed) 9. Which of the following do you believe are true about blood donation? (Checkboxes - Select all that apply) - M1: Donating blood makes you weak - M2: You can catch diseases like HIV from donating blood - M3: Women should not donate blood - M4: You must fast before donating blood - M5: People with tattoos can't donate - None of the above **Section 4: Beliefs and Perceptions** 10. What motivates you the most to donate blood? (Multiple Choice) - Altruism (helping others) - Incentives or rewards - Peer or family encouragement - Personal experiences with someone needing blood - Other 11. If you haven’t donated, what is the biggest reason? (Multiple Choice) - Fear of needles or side effects - Lack of time - Health issues - Religious beliefs - I was never asked - Misinformation or myths - Other *Show only if Q6 = No* **Section 5: Future Intent & Trust** 12. Are you willing to donate blood in the future? (Multiple Choice) - Yes - No - Maybe 13. Where did you first learn about blood donation? (Multiple Choice) - Social media - TV or radio - Posters or flyers - Healthcare professionals - School or university - Friends or family - Religious or community groups 14. How much do you trust the healthcare system to manage blood donation safely and ethically? (Linear Scale: 1 = No trust, 5 = Full trust) **Section 6: Blood Type Information** 15. Do you know your blood type? (Yes/No) 16. If yes, what is your blood type? (Multiple Choice) - A+ - A− - B+ - B− - AB+ - AB− - O+ - O− *Show only if Q15 = Yes* **Final Section (Optional)** 17. Do you consent to your responses being used anonymously for research purposes? (Yes/No) 18. Any comments or suggestions about blood donation or this survey? (Paragraph)
